# Critical aspects in developing curriculum-based assessment for emerging surgical procedures

**DOI:** 10.5116/ijme.61ba.07c6

**Published:** 2021-12-23

**Authors:** Ruth Blackham, Jeffrey Hamdorf

**Affiliations:** 1 Medical School, The University of Western Australia

**Keywords:** Medical education, curriculum, surgery, assessment, simulation

## Introduction

The past century has seen an enormous rise in new surgical procedures. The general surgery landscape has changed markedly, from predominantly open procedures to laparoscopic and now robotic surgery.  Surgical training has been far slower to adjust to change, as evidenced by the haphazard method of introduction of laparoscopic surgery.  The introduction of laparoscopy was marked by procedures being performed by surgeons without a structured fellowship training program, in addition to questionable legitimacy of procedures due to the lack of supportive data.^1^

New surgical methods and new technologies, such as robotic surgery, have been accompanied by a greater emphasis upon educational efforts prior to surgical "practice".  Capabilities such as dual consoles to maximise the teaching potential of the platform in addition to exposure during training lends some recognition to this modern requirement.^2^ Whilst adjuncts such as certified proctorship and simulation exposures have arisen, there remains a need for academic rigour via validity testing to ensure robust and defensible curricula are developed.^3^ This is especially important when the curriculum articulates into an assessment tool with a rating scale and associated rubric.

To date, the most common methods of assessing a surgeon for accreditation by an institution have relied upon surrogate competency measures. The two most common are Years of experience^4^^,^^5^ and Case volume-based measures. 6-8 Both may be problematic, especially when assessing surgeons who wish to undertake new procedures as time in practice do not necessarily equate to transferability of skillset; likewise, volume-based criteria require some degree of overlap of skill from one surgical procedure to another.  The use of simulator-based teaching and assessment shows great promise in its ability to be used for de-identified, reproducible and but the method by which curricular documents and subsequent assessment matrices are developed is not clearly defined.  In this paper, we describe a number of critical considerations in creating credentialing guidelines for surgeons in novel or emerging procedures.

### Novel procedures and emerging technologies

Refinement of existing surgical procedures is ubiquitous by surgeons and device technology developers alike. However, the past half-century has seen an unprecedented rise in new operations. In general surgery, the development of laparoscopy has led to a legion of new ways to perform existing procedures and a paradigm shift in skillset, almost unrecognisable to that of a traditionally-trained "open" surgeon.  In hindsight, the methodology of learning laparoscopic surgery was at best haphazard; at worst, dangerous with a concomitant rise in morbidity and mortality.

Similarly, the introduction of robotic surgery has shown benefits of shortened hospital stays and complication rates, tempered by increased intraoperative times, cost and increased training burden of the new technique.^9^

The belated introduction of structured courses such as Fundamentals of Laparoscopic Surgery course^10^ followed the more routine use of laparoscopy in gynaecology and general surgery, such that it is now the normative approach worldwide. More recently, the introduction of robotic surgery had been accompanied by a greater awareness of the difficulties in introducing surgeons to new technology requiring novel skills and an increased reliance upon simulation in addition to proctorship for training and credentialing.^11^

### Curriculum processes – modified Delphi methodology

Proceduralists benefit from standardised, validated curriculums for teaching competence focused upon specific skills.^12^ There are a number of methods wherein a structured curriculum for teaching a new skill or procedure can be developed.  The most ubiquitous is the "expert opinion" method, with variations upon the rigour with which it is created. The older "hot-tubbing" approach involved a group of experts discussing and creating a curriculum within a set time frame.  The more formal, validated approach requires a defined expert-level opinion formed via an iterative forecasting method via multiple rounds seeking consensus, also known as the Delphi method.  For example, utilisation of the Delphi process to develop a proficiency-based progression course in training for robotic surgery.^13^ Benefits of the Delphi approach include the validation of a formal consensus opinion as well as the statistical significance of reaching consensus via Cronbach's alpha. However, in addition to defining the curriculum, a further demonstration of the use of a skills curriculum over existing teaching paradigms is required.^14^ A second limitation is the lack of a readily accessible group of experts when the technology is only newly developed or a novel surgical procedure wherein the number of experts may not be enough to utilise Delphi methodology^15^; thus, the option of informal expert consensus is likely to continue in future with caveats in place.

### Content validity

The creation of a formal, structured curriculum for a novel surgical procedure must be adjusted to suit the local environment.  The curriculum must be structured to suit local nuances of device availability, preference of technique, and patient demographic. Validating the content of a curriculum towards a particular region is often conducted via Delphi methodology similar to above, with the expert group derived entirely from the defined area.^16^^,^^17^ In doing so, the teaching method and assessment are more relevant for the country or region with greater applicability when extended into the credentialing environment.  However, with greater specificity, there is a loss of generalisability; the potential for bias dependent upon ethnicity and place of qualification must be recognised by the assessor.^18^ Such deficiencies in procedure would need to be noted by the credentialing body.

### Construct validity

One of the most powerful aspects of a validated curriculum is the ability to discern a novice from an expert surgeon. The definitions of these are highly debatable and is most notably determined by surrogate competency markers such as case-volume load or time-based (years in practice).  There is a move towards standardised, reproducible content assessment, which often requires the use of a simulator.  Via direct observation or retrospective video review of a surgical operation upon a simulator, a trained assessor can rate them upon predefined metrics, utilising construct validity to determine whether the assessment score relates to the allocated group of experts or surgeons.  The preferred approach of a simulator removes the requirement for statistical adjustment for patient comorbidities such as body habitus, previous surgery or adhesions.  However, its transferability to "real-life surgery" is an extra consideration that often needs to be validated prior to use as a high-stakes assessment methodology. Defining criteria for credentialing to determine "expert status" utilising consensus methodology has been applied in robotic^19^ and laparoscopic surgery.  Development of learning curve phases from "competent" to "proficient", then "mastery" of procedures^20^ allows for a more nuanced comparison of the level of expertise.

## Conclusions

The determination of a curriculum-based assessment for novel surgical procedures is an exemplary modality of reproducible, quality assessment.  The rise of high-stakes assessment requires greater rigour in the development of the underlying curriculum with which surgeons are being trained to ensure competency upon the simulator as well as ensuring patient safety. Our paper describes the key educational and clinical concerns regarding the development of construct and content validity as well as components of transferability for assessment.  Future assessment of surgeons in novel surgical procedures will likely require credentialing bodies to be cognizant of similar considerations of defensible curricular and assessment development.

### Conflict of Interest

The authors declare that they have no conflict of interest.
